# Linear-Scaling Implementation of Multilevel Hartree–Fock Theory

**DOI:** 10.1021/acs.jctc.1c00299

**Published:** 2021-11-08

**Authors:** Linda Goletto, Eirik F. Kjønstad, Sarai D. Folkestad, Ida-Marie Høyvik, Henrik Koch

**Affiliations:** † Department of Chemistry, Norwegian University of Science and Technology, Trondheim 7491, Norway; ‡ Scuola Normale Superiore, Pisa 56126, Italy

## Abstract

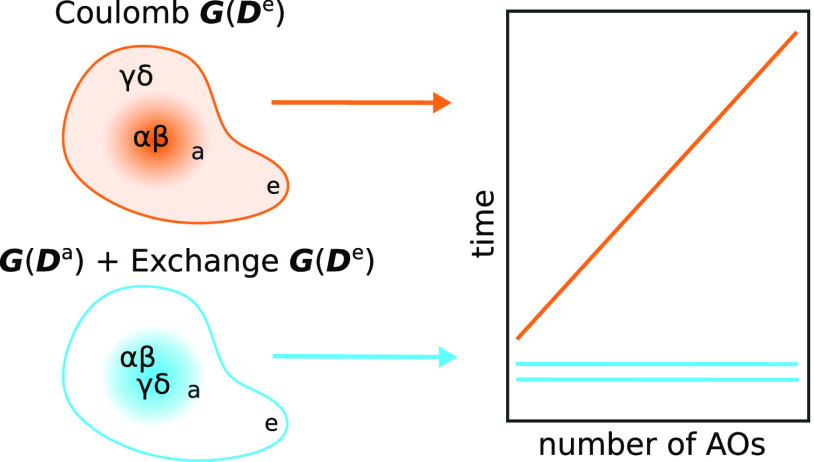

We introduce a new algorithm for the construction of the two-electron contributions to the Fock matrix in multilevel Hartree–Fock (MLHF) theory. In MLHF, the density of an active molecular region is optimized, while the density of an inactive region is fixed. The MLHF equations are solved in a reduced molecular orbital (MO) basis localized to the active region. The locality of the MOs can be exploited to reduce the computational cost of the Fock matrix: the cost related to the inactive density becomes linear scaling, while the iterative cost related to the active density is independent of the system size. We demonstrate the performance of this new algorithm on a variety of systems, including amino acid chains, water clusters, and solvated systems.

## Introduction

The most expensive step in a Hartree–Fock (HF) calculation is typically the construction of the two-electron contributions to the Fock matrix. While the formal scaling is , where *N* is a measure of the system size, it reduces asymptotically to ; only  integrals are non-zero in the limit of large *N*. Furthermore, for sparse density matrices, the number of numerically significant exchange terms is reduced to , even if identifying these terms strictly implies a steeper scaling.^1^ Much effort has been devoted to lower the quadratic scaling of the Coulomb term in the Fock matrix. For sufficiently large *N*, the Coulomb contributions can also be calculated in  time.^2^

One strategy to achieve an  Coulomb matrix is to introduce hierarchies of fine and coarse grains for close and remote interactions, respectively. With the Barnes–Hut method,^3^ the scaling was lowered to , while the continuous fast multipole method (CFMM) of White et al.^2^ was the first scheme to reach linear scaling. Many alternative tree-like algorithms have since been developed, with the main goal of reducing the prefactor.^4,5^ For the exchange term, the focus has been on efficiently identifying the numerically significant exchange integrals. The widely adopted LinK algorithm of Ochsenfeld et al.^1^ presorts the contributing integrals while also incorporating permutational symmetry. Other strategies to further reduce the prefactor have been suggested.^6,7^

An important reduction in the time required by the computation of the two-electron integrals has also been obtained through the density fitting (DF)—or resolution-of-identity (RI)—approximation.^8^ Applied on the Coulomb term first,^9,10^ and later on the exchange component,^11^ this approach approximates the four-center electron repulsion integrals by two- and three-center expressions. The method itself does not scale linearly with respect to the system size, but it has been combined with CFMM^12^ and localized orbitals^13^ to yield an asymptotic  scaling. As an alternative to RI, Cholesky decomposition can be used in the integral approximation.^14,15^

Graphical processing units (GPUs) have also proven to be an important asset in the speed-up of the two-electron integral computation;^16^ the introduction of double precision support has allowed for mixed precision approaches that balance accuracy and GPU performance.^17^

Once the Fock matrix has been constructed, a self-consistent field (SCF) algorithm often performs an  diagonalization step to obtain the next guess for the molecular orbital (MO) coefficients. However, due to the sparsity of the atomic orbital (AO) density matrix, this step can be replaced by an  density optimization.^18−21^ A purification procedure, such as McWeeny’s purification,^22,23^ is used to enforce hermiticity, *N*-representability, and idempotency. A detailed review of linear-scaling SCF methods can be found in Ref (24).

Another strategy to achieve linear-scaling HF is to use fragmentation methods that divide the full space into boxes or monomers.^25^ After the definition of the fragments, an SCF procedure is typically performed on each of them. The interaction between fragments can be accounted for in several ways, such as through overlapping buffer regions around the fragments.^26,27^ When the property of interest is localized in a known region of the system, multiscale and multilevel methods can be used. The rationale behind these techniques is that one can—without loss of accuracy in the targeted property—restrict the most expensive quantum mechanical treatment to an active region of the system. The environment is treated either as a continuum,^28−30^ at a molecular mechanics level,^31−33^ or by using a less expensive quantum mechanical model.^34−41^

The multilevel Hartree–Fock (MLHF) method was introduced by Sæther et al.^42^ This approach bears some resemblance to the local SCF method^43,44^ and is closely related to the QM/ELMO method recently proposed by Macetti and Genoni.^45^

In MLHF, the total density is written as a sum of an active and an inactive density matrix, where only the active density is optimized. Interactions with the environment are included through a constant contribution to the Fock matrix. The MLHF method is designed for systems where the active region is small with respect to the full system size, such as solvated systems or proteins with a well-defined active site. It offers a reliable reference wave function for reduced space coupled cluster calculations of intensive properties, where the correlation treatment is restricted to a set of active MOs.^46−48^

Due to the active–inactive partitioning, the MLHF equations can be solved in the space of the localized active MOs. The cost of diagonalization is therefore independent of the system size. Furthermore, the locality of the MOs can be used to reduce the cost of the AO Fock matrix; several terms do not contribute to the active MO matrix and can be neglected.^42,49^ This fact has, however, only been partially exploited in previous implementations.^42,46,50^

In this article, we present an efficient MLHF Fock matrix algorithm that fully exploits the local nature of the active MOs. The environment density contributions can be calculated at a cost that scales as , while the iterative cost, consisting of active density contributions, is independent of the system size. Our MLHF implementation is based on a conventional direct HF implementation. We emphasize that any improvement in HF algorithms—such as RI or CFMM—can be incorporated into an implementation of the MLHF method.

## MLHF Theory

In MLHF,^42^ the total density matrix is partitioned into an active and an environment (or inactive) density, ***D***^a^ and ***D***^e^
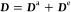
1The active, environment, and total density matrices are required to separately fulfill the hermiticity, trace, and idempotency conditions. The environment density is determined and fixed at the beginning of the calculation, whereas the active density is obtained by minimizing the HF energy.

Using eq 1, with terms given in the AO basis, we can express the HF energy for a closed-shell system as
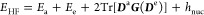
2where
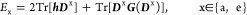
3Here, *h*_nuc_ is the nuclear repulsion energy, ***h*** is the one-electron Hamiltonian integral matrix, and
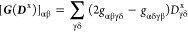
4is the two-electron contribution to the Fock matrix. The two-electron Hamiltonian integrals are denoted as *g*_αβγδ_, where α, β, γ, and δ are AO indices.

The environment density, ***D***^e^, enters the energy minimization through the Fock matrix
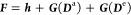
5By projecting the Fock matrix onto the localized MO basis, we obtain a set of MO Roothaan–Hall equations that are solved iteratively to optimize ***D***^a^. Convergence acceleration can be achieved through, for example, direct inversion of the iterative subspace.^49,51,52^ The ***h*** and ***G***(***D***^e^) terms are computed once at the beginning of the calculation and transformed to the current MO basis in every iteration.^42,49^ Therefore, one only needs to accurately represent the two-electron contributions in the active MO basis. In this basis, ***G***(***D***^x^) is given by
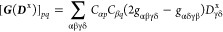
6Here, *p* and *q* refer to MO indices, and ***C*** contains the active MO coefficients.

The active and inactive orbital spaces can be obtained from an idempotent starting guess for the total density. A common starting guess is a superposition of atomic densities^53^ (SAD), ***D***^SAD^. However, ***D***^SAD^ is not idempotent. To fulfill idempotency, ***D***^SAD^ can be used to build a Fock matrix which is then diagonalized.^42^ Due to the sparsity of the SAD guess, which is block-diagonal, this is an  Fock matrix construction with a small prefactor.^53^ Alternatively, it is possible to use a more accurate starting guess, such as a superposition of molecular densities (SMD),^54^ with methods like McWeeny’s purification.^22,23^ The small prefactor of matrix multiplications can make this  procedure advantageous compared to the construction and diagonalization of a Fock matrix.

To determine the initial active occupied orbitals, we perform a restricted partial Cholesky decomposition of the initial idempotent density^55,56^
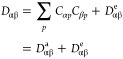
7where the index *p* is restricted to the active occupied MOs. The decomposition is restricted in the sense that pivoting elements are required to correspond to AOs on a set of active atoms.

For the active virtual space, we use projected atomic orbitals (PAOs).^57,58^ The PAOs are generated by projecting out the occupied components (both active and inactive) from the subset of AOs centered on the active atoms, {α̅}
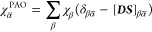
8Since the obtained PAOs are linearly dependent, an orthonormalization procedure, for example Löwdin orthonormalization,^59^ is required to form non-redundant and orthogonal PAOs.

## Linear-Scaling Algorithm for the Fock Matrix

The MLHF Fock matrix has two-electron contributions arising from both the active and the environment density, that is, ***G***(***D***^e^) and ***G***(***D***^a^). The ***G***(***D***^e^) matrix is calculated at the beginning of the calculation and subsequently transformed to the initial active MO basis. In the SCF procedure, ***G***(***D***^e^) is updated to the current MO basis in each iteration through an MO-to-MO basis transformation. In contrast, ***G***(***D***^a^) must be recalculated in every iteration.

The two-electron contributions, and especially ***G***(***D***^e^), have been found to dominate the computational cost in most MLHF calculations.^42,46^ However, in previous implementations of MLHF, these terms were not constructed using sufficiently optimized Fock matrix algorithms. In the original algorithm, which was implemented in a local version of LSDALTON,^60^ the locality of the active MOs was only exploited to truncate the AO basis: the AOs that did not contribute to any of the active MOs were discarded at the beginning of the calculation. This screening algorithm, since it only considers contributions to the MOs, does not exploit all the information available when constructing specific Fock matrix elements. While the algorithm reduces the asymptotic scaling, it was found to be ineffective, except for very large systems.^42^

The implementation in *e^T^* 1.0,^46^ on the other hand, relied on a specialized Fock matrix algorithm which made use of the MO coefficients to skip negligible contributions to ***G***(***D***^a^). However, while this reduced the iterative cost, it did not strictly change the scaling of the underlying Fock construction algorithm. It also did not apply screening to the construction of ***G***(***D***^e^),^46^ thus making the non-iterative cost higher than necessary.

The scaling of ***G***(***D***^e^) and ***G***(***D***^a^) can be reduced to  and  by fully exploiting the local nature of the active MOs. This reduced scaling is readily understood by considering the restriction of the AO indices to active and inactive sets, as implied by the ***G***(***D***^x^) expression in eq 6. Here, we define the set of *active* AOs as the AOs that contribute to the active MOs, that is, the AOs that correspond to significant elements in the active MO coefficients. Note that these active AOs are not only centered on the active atoms but can also belong to atoms in the inactive region that are close to the active atoms. Similarly, we define the set of *inactive* AOs as those that contribute to the environment density. The sets of active and inactive AOs overlap.

Since the coefficients *C*_α*p*_ and *C*_β*q*_ in eq 6 refer to the active set of MOs, only active α and β (in the sense defined above) will contribute to ***G***(***D***^x^). In the case of ***G***(***D***^a^), the γ and δ indices in eq 6 are also active due to the *D*_γδ_^a^ factor. All the AO indices (α, β, γ, and δ) are thus active, and so the cost of ***G***(***D***^a^) will be .

**Figure 1:**
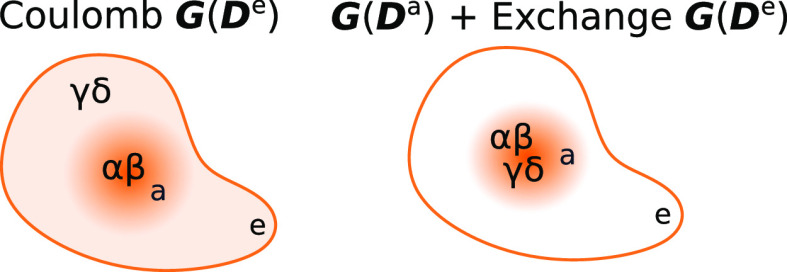
Localization of the AO indices in the Coulomb and exchange contributions. In the Coulomb contribution to ***G***(***D***^e^), the α and β indices are active, in the sense that they contribute to the active MOs, whereas γ and δ are inactive. For ***G***(***D***^a^), and the exchange contribution to ***G***(***D***^e^), all AO indices are active.

For ***G***(***D***^e^), the Coulomb and exchange terms must be considered separately. In the Coulomb contribution
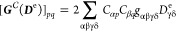
9the γ and δ indices are inactive, but they are also located on atoms separated by a small distance; otherwise *g*_αβγδ_ would be zero. The number of surviving pairs γδ, and consequently the cost of , therefore scales as . On the other hand, the exchange contribution
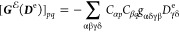
10can be calculated as  because δ and γ are close to the active indices α and β, respectively; otherwise *g*_αδγβ_ would be zero. The localization of the AO indices in the various two-electron terms is depicted in Figure 1.

The ***G***(***D***^e^) term can be computed once in the beginning of the MLHF calculation at an  cost. The iterative cost of MLHF is dominated by the  construction of ***G***(***D***^a^). The scaling is reduced by at least one order compared to conventional HF, where the Coulomb and exchange terms have a quadratic and linear-scaling cost, respectively.

The index restrictions required to efficiently calculate these terms can be determined in a prescreening procedure. In our implementation, lists of significant shell pairs are prepared prior to entering the construction loop for the two-electron contribution to the Fock matrix. These lists are shell-based, instead of AO-based, because the integrals are computed in shell batches by Libint 2.^61^ Prescreening allows us to avoid looping over negligible terms when calculating the two-electron contributions, thereby ensuring the correct scaling.

The screening algorithm is designed to calculate contributions to the MO Fock matrix to a given precision. The algorithm is based on the observation that an element of the AO matrix can be neglected when all contributions to the corresponding MO matrix are below some specified threshold
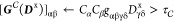
11
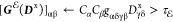
12Here, *C*_α_ = max_*p*_|*C*_α*p*_|, and  and  are the Coulomb and exchange thresholds, respectively. The magnitude of the integrals is estimated using the Cauchy–Schwarz inequality
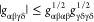
13For compatibility with the integral program,^61^ these conditions are implemented for shells rather than individual AOs. When expressed in terms of AO shells {*s*_*i*_}, and with Cauchy–Schwarz estimates for the integrals, the conditions in eqs 11 and 12 become
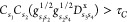
14
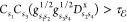
15where we have defined the shell-based quantities
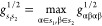
16
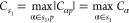
17
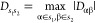
18In the following, we will also make use of the quantities
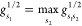
19
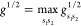
20
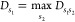
21
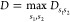
22
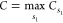
23

The active MOs determine which ***G***(***D***^x^) contributions are negligible. When the screening is applied to ***G***(***D***^a^), we always use the current active MOs. On the other hand, when it is applied to ***G***(***D***^e^), we use the initial active MOs. As a result, the introduced error in ***G***(***D***^e^) is proportional to, and not bounded by, the threshold. In practice, it is sufficient to use the same thresholds without a significant loss of accuracy.

The screening conditions in eqs 14 and 15 assume information about the four shells *s*_1_, *s*_2_, *s*_3_, and *s*_4_, which is only available in the inner-most loop of a Fock matrix construction. An efficient implementation, however, must exploit the information available at any given level of the nested loop. This is accomplished using a set of looser screening conditions, derived from eqs 14 and 15, where all information available at a given level is used to screen out negligible terms.

The procedures used to calculate the Coulomb and exchange terms are given in algorithms 1 and 2. In both algorithms, the first step is to determine the set of shell pairs *s*_1_*s*_2_ that correspond to non-negligible two-electron integrals. The significant shell pair list
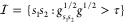
24is prepared at the beginning of the MLHF calculation. Here, τ is an integral cutoff threshold, while  and *g*^1/2^ are defined in eqs 16 and 20, respectively. In the outermost loop, over the *s*_1_*s*_2_ in , we can use screening conditions derived from eqs 14 and 15 for the given *s*_1_ and *s*_2_ (see line 3 of algorithms 1 and 2). Note that these conditions also take into account permutational symmetry. A shortened list of significant shell pairs *s*_1_*s*_2_ () is thus constructed, in addition to a list of the significant *s*_1_ () and a list of significant *s*_2_ for each *s*_1_ (). The dimensions of , , and  all scale linearly with the size of the system for  and are constant for  and ***G***(***D***^a^). This prescreening step is detailed in lines 2–9 of algorithms 1 and 2.
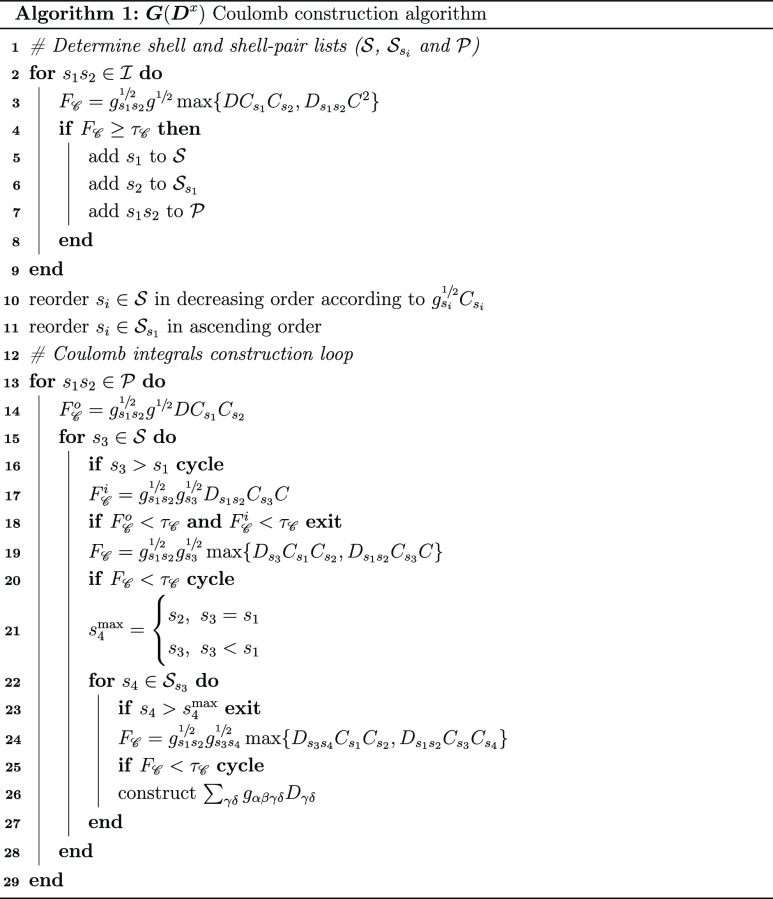


The elements of  are ordered in different ways for  and . To allow for an early exit in the  algorithm, the ordering follows the magnitude of the  products. In the  case, the desired scaling is already achieved, and  is sorted in the ascending order. The  list is ordered according to ascending *s*_2_ to efficiently exploit permutational symmetries (see lines 23 and 19 in algorithms 1 and 2).
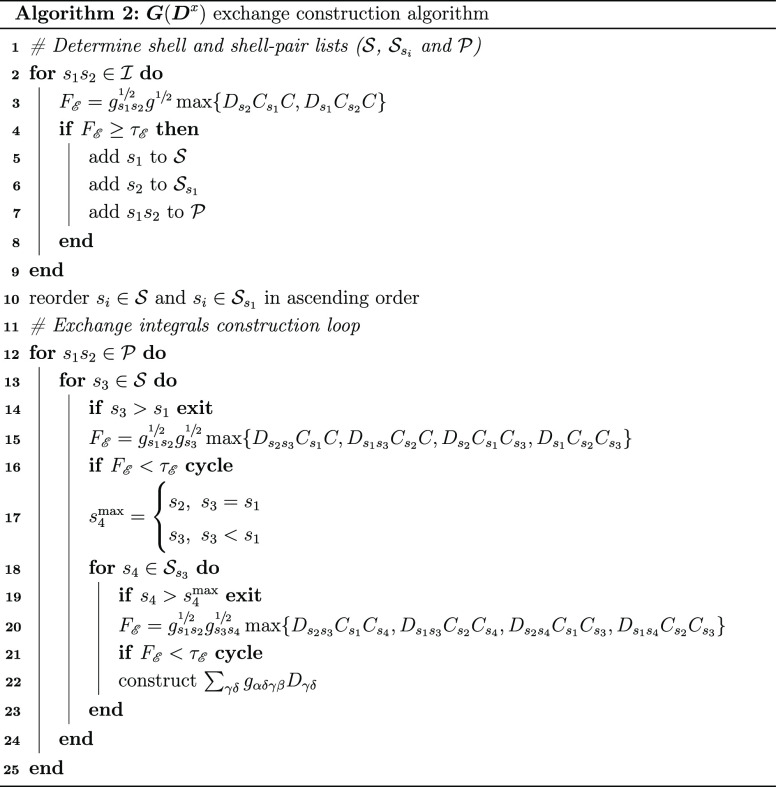


In the construction loop for  and , we first loop over  and . At this point, we can formulate screening criteria, from eqs 14 and 15, for the given *s*_1_, *s*_2_, and *s*_3_. These criteria are used to either exit the *s*_3_ loop or to cycle to the next *s*_3_; see lines 14 and 17–20 in algorithm 1 and lines 15–16 in algorithm 2. When the inner-most *s*_4_ loop is reached, all the shells are known. Therefore, the Coulomb and exchange conditions in eqs 14 and 15 can be used, though some minor modifications are required to account for permutational symmetry; see lines 24–25 in algorithm 1 and lines 20–21 in algorithm 2.

It is also possible to compute  and  in the same construction loop. In this case, we use the structure in algorithm 1, but the exchange conditions given in algorithm 2 are added in the corresponding loops.

Our discussion so far has focused on the scaling of the ***G***(***D***^x^) construction loops. In general, the prescreening steps scale more steeply. In both algorithms 1 and 2, the prescreening loop scales linearly with the system size. In the case of , the reordering scales as , while it is independent of the system size for  and ***G***(***D***^a^). Furthermore, some of the quantities in eqs 16–23 have a cost that scales quadratically, albeit with small prefactors. However, for the systems we are targeting (10^3^ to 10^5^ AOs), their cost is negligible when compared to the cost of constructing the Fock matrix.

An overview of the computational scaling of terms related to ***G***(***D***^x^) is given in Table 1; in particular, the table shows the effects of the ***C***-screening. Furthermore, it presents the scaling of the prescreening lists *g*^1/2^ and *D*, as well as terms related to the construction of the SAD Fock matrix.

**Table 1:** Computational Scaling of Terms in the MLHF Implementation, with and without Screening with Respect to the MOs (***C***-Screening)

	computational scaling		
step	no ***C***-screening	***C***-screening	
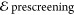	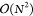	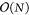	iterative
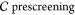	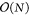	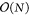	iterative
reordering ()		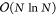	iterative
reordering (, ***G***(***D***^a^))		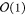	iterative
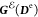	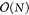	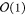	non-iterative
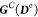	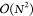	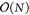	non-iterative
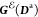	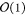	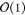	iterative
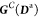	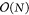	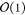	iterative
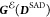	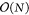		non-iterative
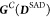	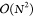		non-iterative
*g*^1/2^ list	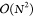		non-iterative
*D* lists	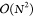		iterative

There are additional steps which may scale more steeply than the terms in Table 1. At the beginning of the MLHF calculation, linear dependence is eliminated from the AO basis by  Cholesky decomposition (or, alternatively, by diagonalization) of the overlap matrix. The one-electron Hamiltonian integrals are also computed at this stage; this  step has a small prefactor and can be made linear with the same multipole strategies that have been developed for the Coulomb matrix in HF theory.^24^ These non-iterative steps are the same as in standard HF. The MLHF procedure also includes a non-iterative step to determine the initial active orbitals, a procedure which is  scaling.

In addition to the cost of ***G***(***D***^a^), and the related prescreening steps, the iterative cost of MLHF includes the cost of adding the elements [***G***(***D***^a^)]_αβ_ to the AO Fock matrix, as well as the subsequent AO-to-MO transformation. These steps are  scaling processes. The Roothaan–Hall optimization is performed in the MO basis and therefore does not entail any steps that scale with the size of the system. The initial Roothaan–Hall diagonalization of the SAD Fock matrix, however, is performed in the AO basis and is therefore an  step. However, for the systems we are targeting, the computational cost is invariably dominated by the construction of ***G***(***D***^e^) and ***G***(***D***^SAD^).

OpenMP parallelization is applied to the outer index *s*_1_*s*_2_ of the main construction loops in algorithms 1 and 2. Each thread can either have its own copy of the Fock matrix or add calculated contributions to a shared copy. With a copy for each thread, one avoids the overhead resulting from threads having to wait for access to memory locations. The memory penalty of keeping a copy for each thread may become a bottleneck for sufficiently large systems. One approach to remove this memory bottleneck is to have a number of threads share a copy of the Fock matrix.^62^ An alternative is to compress the Fock matrix,^63^ so that every thread can hold a copy.

In the MLHF approach, the selection of the significant elements for the compressed Fock matrix can be performed using the same screening conditions applied in algorithms 1 and 2. This results in an asymptotically non-scaling memory requirement for the copies of the Fock matrix in MLHF. In HF, on the other hand, the memory requirement is asymptotically linear with respect to the system size when the density matrix is sparse. In this paper, compression is adopted when the memory requirement becomes a limiting factor.

## Results and Discussion

Algorithms 1 and 2 have been implemented in a development version of the *e^T^* program.^46^ We use a Cholesky decomposition to obtain the occupied orbital space and PAOs to obtain the virtual active MOs. A threshold of 10^–1^ is used for the Cholesky decomposition. In all calculations, we apply a gradient threshold of 10^–6^, giving default values for  and  equal to 10^–12^ and 10^–10^, respectively. The different thresholds are all expressed in atomic units.

Unless otherwise stated, the initial idempotent density guess is obtained from SAD through a diagonalization of the corresponding Fock matrix.

All geometries can be found in ref (64), and we use UCSF Chimera^65^ to visualize them.

### Scaling Properties

**Figure 2:**
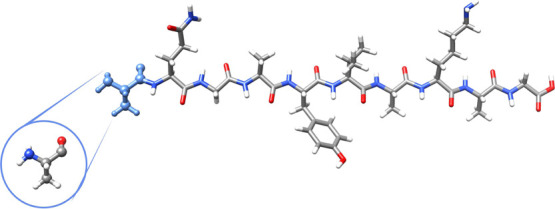
Shortest amino acid chain used in our calculations. The active atoms (those of the alanine at the N-terminal of the chain) are highlighted in blue.

**Figure 3:**
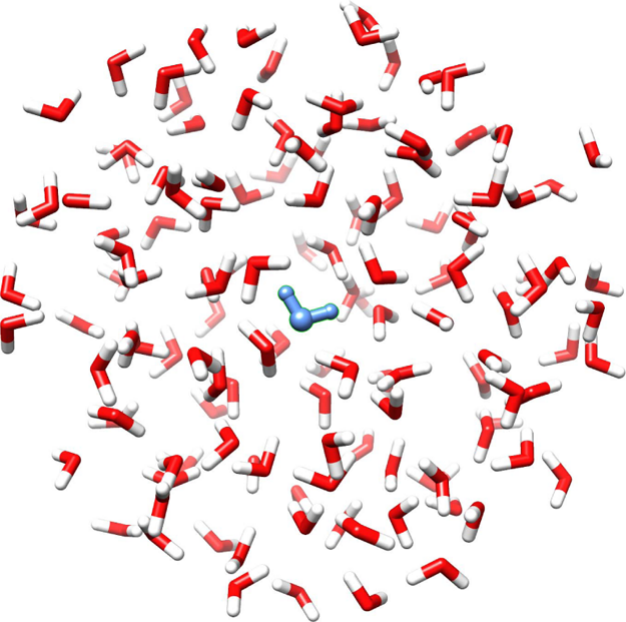
Smallest cluster of water molecules used in our calculations. The active water molecule is highlighted in blue.

The scaling properties of the implementation are demonstrated on two sets of model systems: linear chains of amino acids, constructed by repeating the unit shown in Figure 2, and water clusters of increasing radius, the smallest of which is shown in Figure 3.

**Figure 4:**
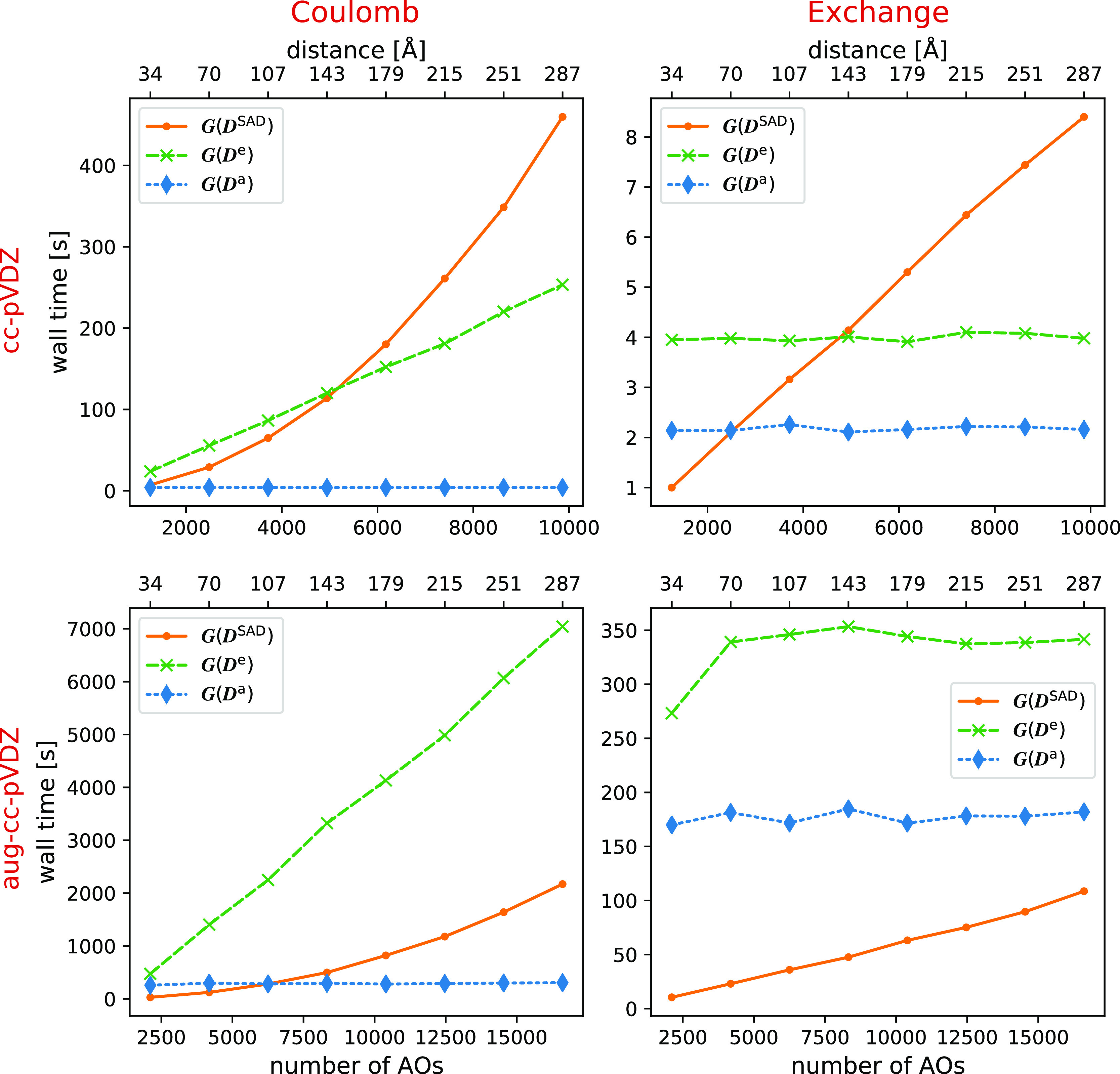
Wall time for MLHF calculations on the linear amino acid chains, with ***C***-screening. All calculations were performed on two Intel Xeon-Gold 6138 processors with 20 cores each with 160 GB memory available.

For the amino acid chain, we define the alanine at the N-terminal as active and use both the cc-pVDZ and aug-cc-pVDZ basis sets. The timings for the Coulomb and exchange contributions to ***G***(***D***^SAD^), ***G***(***D***^e^), and ***G***(***D***^a^) are given in Tables 2 and 3 and depicted in Figure 4. The tables highlight the improvement in the scaling due to the ***C***-screening. Without the ***C***-screening, the active density reduces the scaling by a factor of *N*, but the information in the active MO coefficients is not exploited. This results in  scaling linearly with the size of the system, while  is independent of the system size. For ***G***(***D***^e^), since the density is not localized to the active shells, the scaling is the same as in a general Fock matrix construction, that is, for non-***C***-screened algorithm, the Coulomb term scales quadratically and the exchange term scales linearly. The results in Tables 2 and 3 and Figure 4 show that the ***C***-screening implementation reduces the costs for all two-electron contributions to the Fock matrix and reduces the scaling for  and ***G***(***D***^e^).

**Table 2:** Wall Time for MLHF/cc-pVDZ Calculations on the Linear Amino Acid Chains^a^

	no ***C***-screening						***C***-screening					
	***G***(***D***^SAD^)		***G***(***D***^e^)		***G***(***D***^a^)		***G***(***D***^SAD^)		***G***(***D***^e^)		***G***(***D***^a^)	
#AOs	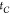	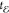	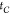	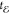	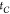	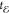	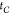	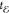	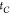	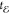	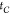	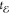
1254	7	1	54	29	22	7	7	1	24	4	4	2
2484	29	2	226	75	51	7	29	2	56	4	4	2
3714	65	3	508	119	79	7	65	3	86	4	4	2
4944	115	4	887	175	107	7	114	4	120	4	4	2
6174	179	5	1448	208	134	7	180	5	152	4	4	2
7404	273	6	2040	249	166	7	261	6	181	4	4	2
8634	352	8	2797	300	193	7	348	7	220	4	4	2
9864	470	9	3657	336	220	7	460	8	253	4	4	2

a The timings are expressed in seconds without and with ***C***-screening. All calculations were performed on two Intel Xeon-Gold 6138 processors with 20 cores each with 160 GB memory available.

**Table 3:** Wall Time for MLHF/aug-cc-pVDZ Calculations on the Linear Amino Acid Chains^a^

	no *C*-screening						*C*-screening					
	***G***(***D***^SAD^)		***G***(***D***^e^)		***G***(***D***^a^)		***G***(***D***^SAD^)		***G***(***D***^e^)		***G***(***D***^a^)	
#AOs	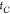	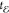	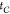	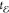	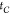	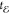	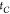	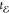	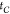	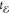	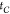	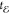
2112	28s	10s	10	8	8	5	29s	10s	8	5	4	3
4183	2	22s	44	27	23	8	2	23s	23	6	5	3
6254	5	37s	102	46	36	8	5	36s	37	6	5	3
8325	8	49s	179	66	50	8	8	48s	55	6	5	3
10396	13	1	282	87	63	8	14	1	69	6	5	3
12467	19	1	411	109	79	8	20	1	83	6	5	3
14538	27	1	562	133	96	8	27	1	101	6	5	3
16609	36	2	754	150	112	8	36	2	117	6	5	3

a The timings are expressed in minutes when not stated otherwise, without and with ***C***-screening. All calculations were performed on two Intel Xeon-Gold 6138 processors with 20 cores each with 160 GB memory available.

As mentioned before, the ***C***-screening, like all screening methods based on the overlap of orbitals, performs better with non-diffuse basis sets. However, these results show that the  scaling can be reached with both basis sets. The wall time for the prescreening steps and for some relevant non-iterative procedures in the calculations is reported in the Supporting Information.

The calculations on the amino acid chains illustrate the behavior of the algorithm for a one-dimensional system. Since many systems of interest are three-dimensional, we also consider the scaling properties on water clusters where the central water molecule is active. Several combinations of basis sets have been selected; in the following, the notation *x*/*y* (e.g., aug-cc-pVDZ/STO-3G) is used to denote that the active water molecule is treated with the basis *x* and the environment with the basis *y*.

**Figure 5:**
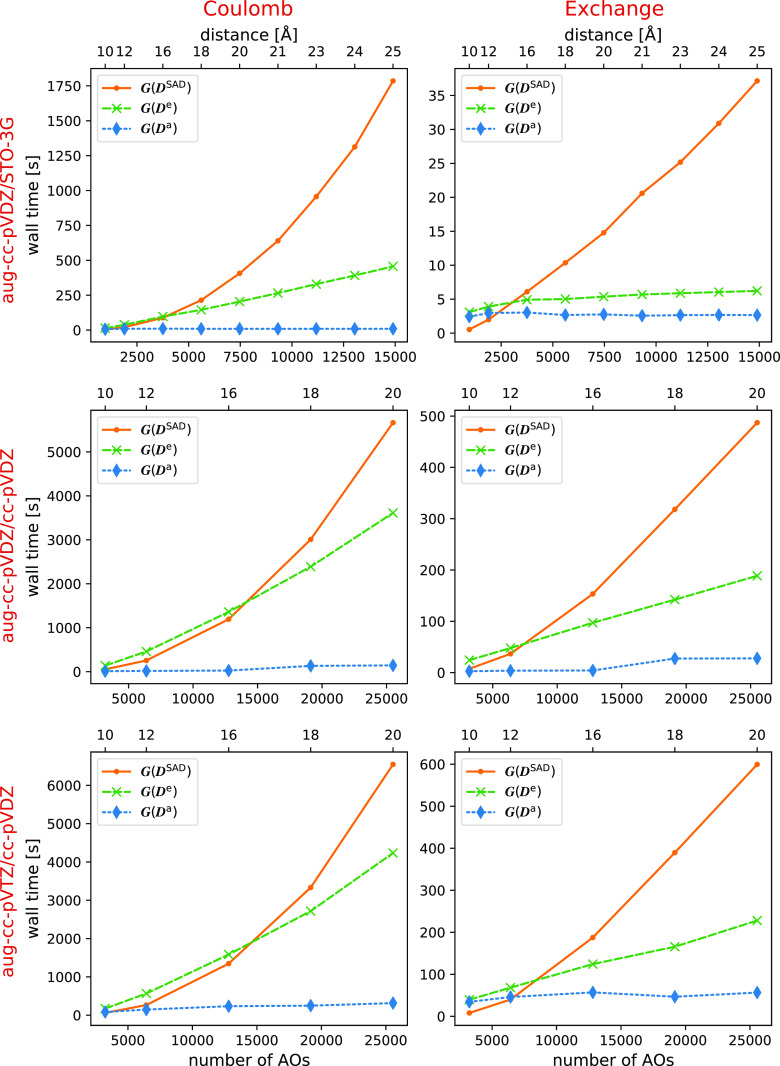
Wall time for MLHF calculations on clusters of water molecules of increasing radius, with ***C***-screening. All calculations were performed on two Intel Xeon-Gold 6138 processors with 20 cores each. The calculations with the environment treated with STO-3G were given 160 GB memory; the calculations with a cc-pVDZ environment were performed with 360 GB memory available.

Wall time for aug-cc-pVDZ/STO-3G calculations is shown in the first row of Figure 5. When the environment is treated with a minimal basis, the calculations rapidly exhibit the correct scaling, even if diffuse basis functions are used on the active atoms. This may be of some practical importance since the active atoms must have diffuse functions for correlated methods to predict intensive properties with quantitative accuracy. Furthermore, an adequate frozen environment density may not require a high-quality basis set.

In the last two rows of Figure 5, we report the wall time with the aug-cc-pVDZ/cc-pVDZ and aug-cc-pVTZ/cc-pVDZ basis set combinations. The computational cost of the  and  terms is approximately constant with respect to the cluster size. On the other hand, the  term has a scaling in-between  and , and the  term scales as . The observed scaling is thus different from the asymptotic scaling of these terms. Due to the larger number of AOs per atom, these are calculations on smaller water clusters than those with the STO-3G environment. Hence, these calculations show that one must extend the environment further to reach the asymptotic scaling. Despite this, the time to construct ***G***(***D***^e^) still becomes smaller than the time required to construct ***G***(***D***^SAD^) when the system exceeds 15 000 AOs. The non-iterative cost is therefore dominated by the ***G***(***D***^SAD^) in the largest systems. Tables with the wall time are given in the Supporting Information.

### Comparison to HF

The MLHF method has already been shown to be significantly cheaper than standard HF.^42,46^ The ***C***-screening detailed in algorithms 1 and 2 reduces the cost and scaling of MLHF even further.

We illustrate these savings by performing MLHF and HF calculations on the system shown in Figure 3, treated with the aug-cc-pVTZ/cc-pVDZ basis set combination. The wall time for the ***G***(***D***^x^) terms (*t*^x^), which completely dominate the corresponding Fock matrix constructions, is given in Table 4. The total wall time for the full calculations, *t*^tot^, is also reported. Compared to the MLHF implementation without ***C***-screening, the total wall time *t*^tot^ is reduced by approximately a factor of 3. With respect to standard HF, *t*^tot^ is reduced by approximately a factor of 5. In particular, the ***C***-screening reduces *t*^e^ by a factor of 2.5 and *t*^a^ by a factor of 4 for ***G***(***D***^a^). The timings for ***G***(***D***^SAD^) are reported for reference, but are, as expected, the same in the three calculations.

**Table 4:** Wall Time, Expressed in minutes, for MLHF and HF Calculations on a Water Cluster with a 10 Å Radius^a^

method	*t*^SAD^ (min)	*t*^e^ (min)	*t*^a^ (min)	*t*^tot^ (min)
MLHF ***C***-screening	1	4	2	12
MLHF no ***C***-screening	1	10	8	40
HF	1		10	57

a The times to construct ***G***(***D***^SAD^), ***G***(***D***^e^), and ***G***(***D***^a^) of the first iteration are denoted as *t*^SAD^, *t*^e^, and *t*^a^. *t*^tot^ is the total wall time of the full calculation. The aug-cc-pVTZ/cc-pVDZ combination of basis sets is used, and there are 3236 AOs. The calculations were performed on two Intel Xeon Gold 6152 processors, with 44 threads and 1.4 TB memory available.

It should be emphasized that the computational savings compared to non-screened MLHF and standard HF depend on the basis set. In particular, the addition of diffuse functions to the basis set has a significant impact on the screening. Although the screening becomes effective at a sufficient distance from the active region, this distance may be quite large. For large basis sets with many diffuse functions, other strategies—for example, RI or Cholesky decomposition—could be incorporated into an MLHF implementation.

### Validating the Screening Algorithm with CC2 Excitation Energies

Our implementation applies ***C***-screening on both active and inactive electron repulsion terms. In this section, we demonstrate that the results are insensitive to the use of the ***C***-screened MLHF wave function as a reference in post-HF calculations of intensive properties.

**Figure 6:**
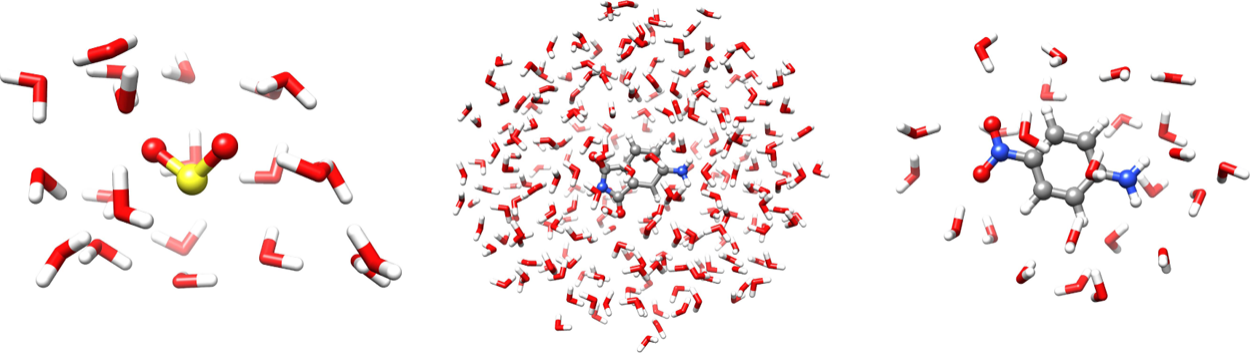
Three solvated moieties—SO_2_, 4-aminophthalimide, and *para*-nitroaniline—treated at the CC2-in-MLHF/HF level. The solute is active in MLHF.

We present CC2 excitation energies of different moieties in aqueous solution, obtained with and without ***C***-screening. The systems—SO_2_, 4-aminophthalimide, and *para*-nitroaniline in water—are depicted in Figure 6. In all cases, the solute is chosen as active and treated with aug-cc-pVDZ, while the surrounding water molecules are treated with cc-pVDZ. Table 5 shows that the ***C***-screening does not affect the computed excitation energies.

**Table 5:** CC2-in-MLHF/HF Excitation Energies, Obtained Using aug-cc-pVDZ on the Active Atoms and cc-pVDZ on the Inactive Atoms

	***C***-screening (eV)	no ***C***-screening (eV)
SO_2_ + water	3.236	3.236
4-aminophthalimide + water	3.845	3.845
*para*-nitroaniline + water	4.036	4.036

### Density Purification and Memory Compression for Large Systems

For large systems, the memory required to keep a copy of the AO Fock matrix for each OpenMP thread can become impractical. Additionally, the ***G***(***D***^SAD^) construction can become the bottleneck since it scales as  with a significant prefactor. To avoid the ***G***(***D***^SAD^) step and the diagonalization of the corresponding Fock matrix, we make use of McWeeny’s purification^22,23^ on an SMD starting guess.^54^ The memory usage for ***G***(***D***^e^) and ***G***(***D***^a^) is reduced by applying compression^63^ to the copies of the Fock matrix.

**Figure 7:**
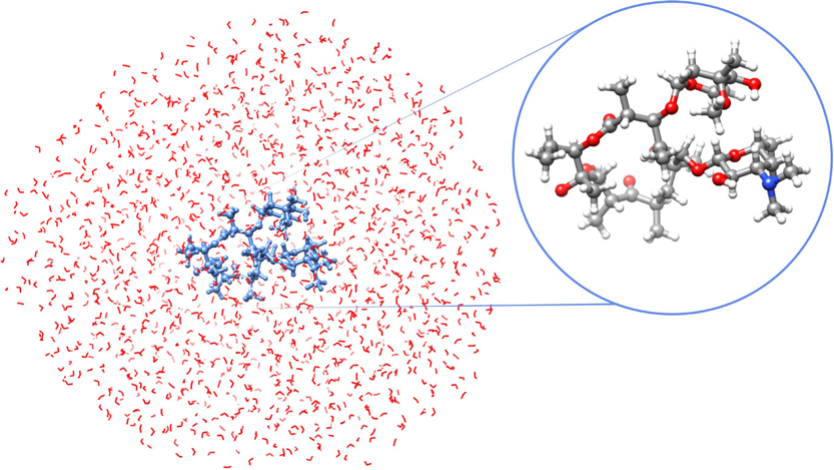
Smallest erythromycin-in-water system used in our calculations. The active erythromycin molecule is highlighted in blue.

We use these strategies on erythromycin-in-water systems, treated with aug-cc-pVTZ/cc-pVDZ. The smallest system, with 42 119 AOs, is depicted in Figure 7. In Table 6, we report timings for the SMD guess *t*^SMD^, the purification *t*^pur^, the memory compression *t*^com^, and the , , and ***G***(***D***^a^) terms, along with the required memory  of a single copy of the compressed matrices. Note that the calculations were carried out on two different machines (A and B), so that the timings cannot be directly compared. The compression scheme entails a computational penalty; however, it makes it possible to reach systems with more than 10^5^ AOs.

**Table 6:** Wall Time and Memory Requirements for MLHF/aug-cc-pVTZ/cc-pVDZ Calculations on a Series of Erythromycin-in-Water Systems^a^

				***G***(***D***^e^)					***G***(***D***^a^)		
	#AOs	*t*^SMD^	*t*^pur^	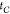	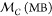	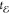	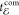	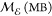	*t*	*t*^com^	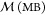
A	42 119	25	1	23	74	5	11	623	9	6	676
	62 111	26	3	33	74	5	12	619	9	6	672
B	82 103	27	9	51	76	6	15	644	12	8	697
	102 119	28	17	64	76	5	15	644	11	8	697

a The timings are expressed in hours, while the memory is given in megabytes. The two calculations with 42 119 and 62 111 AOs were performed on machine A, with two Intel Xeon Gold 6138 processors and 40 threads, while the two calculations with 82 103 and 102 119 AOs were performed on machine B, with two Intel Xeon-Gold 6130 processors and 64 threads.

The cost of memory compression for the exchange term is non-negligible. However, this compression step does not scale with the system size. The cost is mainly due to the lack of OpenMP parallelization. The calculations are still dominated by the Coulomb term. Timings for the Coulomb compression step are not reported as it requires less than a minute in all calculations. This compression scales as  for , and as  for , so its cost will always be negligible compared to other terms.

Due to the need to hold in memory some *N*_AO_^2^ matrices, the memory requirement of the full calculation scales quadratically; in the largest system, a peak memory usage of 518 GB was reached. The memory usage for the compressed Fock matrices is small and scales as  with the system size.

From Table 6, we see that the cost of the SMD construction is significant. It is dominated by the HF calculation on erythromycin. While solvated systems are trivially separated into subsystems, large covalently bound systems require a fragmentation procedure. This would also reduce the cost of SMD for erythromycin-in-water.

## Summary and Concluding Remarks

We have introduced a new algorithm for the two-electron contributions to the Fock matrix in the MLHF method. This algorithm exploits the locality of the active MOs to efficiently screen contributions to the active MO Fock matrix. We achieve  scaling for the construction of  and  scaling in the  and ***G***(***D***^a^) terms. Although the MLHF implementation includes steps that scale more steeply, the Fock matrix construction dominates the iterative and overall costs of calculations on systems with up to 10^4^ to 10^5^ AOs.

To demonstrate the scaling of the implementation, we have presented a number of calculations on one- and three-dimensional systems of increasing size. The efficiency of the implementation was also tested on a water cluster, which provides an illustration of the savings relative to non-screened MLHF and HF. Our algorithm involves additional screening based on the MOs with respect to previous algorithms. We have therefore tested its accuracy by performing excited-state CC2 calculations.

Since the memory required to hold a copy of the AO Fock matrix for every OpenMP thread increases as , the memory usage can become the limiting factor for large systems. At the same time, in these systems the  SAD Fock matrix construction dominates the computational cost. We have therefore combined the two-electron integrals screening with memory compression of the Fock matrix^63^ and McWeeny’s purification^22,23^ of an SMD starting guess,^54^ in order to reach larger system sizes. Calculations on erythromycin-in-water systems with up to 100 000 basis functions have been performed.

In the limit of large *N*, the cost to construct ***G***(***D***^e^) becomes effectively independent of the system size. This is because of the long-range decay of the Coulomb interactions, which is used in HF theory to reduce the asymptotic Coulomb matrix scaling from  to .^24^ For the Coulomb contribution of ***G***(***D***^e^), the  scaling similarly reduces to . This is not to say that *all* costs are independent of the system size: as in other Fock construction algorithms, there may be preparation steps that scale more steeply. Possible further improvements could include an adaptation of the well-established CFMM method,^2^ as well as a combination of the MLHF approach with DF^8^ or Cholesky decomposition.^14,15^

## Supporting Information Available

Wall time comparison for the prescreening procedures of MLHF/cc-pVDZ calculations and MLHF/aug-cc-pVDZ calculations; wall time for non-iterative procedures of MLHF/cc-pVDZ calculations and MLHF/aug-cc-pVDZ calculations on the linear amino acid chains; wall time comparison for the MLHF/aug-cc-pVDZ/STO-3G calculations; and wall time comparison for the MLHF/aug-cc-pVDZ/cc-pVDZ calculations on water clusters of increasing radius (PDF)
