# Supplementary material for: Linear-Scaling Implementation of Multilevel Hartree–Fock Theory
Source: J Chem Theory Comput. 2021 Nov 8;17(12):7416–27. doi: 10.1021/acs.jctc.1c00299 (PMC8675138; doi:10.1021/acs.jctc.1c00299)
Supplement: Supplementary file 1 — ct1c00299_si_001.pdf [file ct1c00299_si_001.pdf]

Supporting information for "*Linear-scaling implementation of multilevel Hartree-Fock theory*"

Linda Goletto,† Eirik F. Kjønstad,† Sarai D. Folkestad,† Ida-Marie Høyvik,† and Henrik Koch*,†,‡

†*Department of Chemistry, The Norwegian University of Science and Technology, Trondheim, Norway*

‡*Scuola Normale Superiore, Pisa, Italy*

E-mail: henrik.koch@sns.it

Tables 1 and 2 present the wall times for the pre-screening steps of the calculations on the amino acid chains. Tables 3 and 4 show the time walls for some significant non-iterative procedure on the amino acid chains. The calculations were performed on two Intel Xeon-Gold 6138 processors with 20 cores each with 160 GB memory available. Tables 5, 6 and 7 present the wall times for the water clusters calculations. The calculations were performed on two Intel Xeon-Gold 6138 processors with 20 cores each with either 160 GB (Table 5) or 360 GB (Tables 6 and 7) memory available. The gradient threshold has been set to 10^{-6} in all calculations, giving default values for $\tau_{\mathscr{C}}$ and $\tau_{\mathscr{E}}$ equal to 10^{-12} and 10^{-10}, respectively.

Table 1: Wall time comparison for the pre-screening procedures of MLHF/cc-pVDZ calculations on the linear amino acid chains, expressed in seconds, without and with C-screening.

	no $\boldsymbol{C}$-screening			$\boldsymbol{C}$-screening		
#AOs	$t_{\text{pre}}^{\text{SAD}}$	$t_{\text{pre}}^{\boldsymbol{G}(\boldsymbol{D}^{\text{e}})}$	$t_{\text{pre}}^{\boldsymbol{G}(\boldsymbol{D}^{\text{a}})}$	$t_{\text{pre}}^{\text{SAD}}$	$t_{\text{pre}}^{\boldsymbol{G}(\boldsymbol{D}^{\text{e}})}$	$t_{\text{pre}}^{\boldsymbol{G}(\boldsymbol{D}^{\text{a}})}$
1254	0.04	0.03	0.03	0.04	0.04	0.03
2484	0.1	0.1	0.1	0.1	0.1	0.1
3714	0.2	0.2	0.3	0.2	0.1	0.1
4944	0.4	0.3	0.4	0.4	0.2	0.2
6174	0.6	0.6	0.6	0.5	0.3	0.3
7404	0.8	1.0	0.9	0.8	0.4	0.4
8634	1.1	1.1	1.2	1.2	0.6	0.6
9864	1.3	1.4	1.5	1.4	0.8	0.7

Table 2: Wall time comparison for the pre-screening procedures of MLHF/aug-cc-pVDZ calculations on the linear amino acid chains, expressed in seconds, without and with C-screening.

	no $\boldsymbol{C}$-screening			$\boldsymbol{C}$-screening		
#AOs	$t_{\text{pre}}^{\text{SAD}}$	$t_{\text{pre}}^{\boldsymbol{G}(\boldsymbol{D}^{\text{e}})}$	$t_{\text{pre}}^{\boldsymbol{G}(\boldsymbol{D}^{\text{a}})}$	$t_{\text{pre}}^{\text{SAD}}$	$t_{\text{pre}}^{\boldsymbol{G}(\boldsymbol{D}^{\text{e}})}$	$t_{\text{pre}}^{\boldsymbol{G}(\boldsymbol{D}^{\text{a}})}$
2112	0.1	0.1	0.1	0.1	0.1	0.1
4183	0.4	0.3	0.4	0.4	0.3	0.2
6254	0.8	0.6	0.8	0.9	0.5	0.4
8325	1.4	1.1	1.5	1.4	0.8	0.7
10396	2.2	1.8	2.3	2.2	1.1	1.0
12467	3.2	2.5	3.3	3.1	1.5	1.3
14538	4.4	3.8	4.5	4.4	1.9	1.8
16609	5.5	5.4	7.0	6.7	2.5	2.5

Table 3: Wall time for non-iterative procedures of MLHF/cc-pVDZ calculations on the linear amino acid chains, expressed in seconds.

#AOs	$t^{S\text{ Cholesky}}$	$t^{F^{\text{SAD}}\text{ diagonalization}}$	$t^{\text{active MOs}}$
1254	0.1	0.4	0.03
2484	0.4	1.5	0.1
3714	1.0	4.0	0.1
4944	2.0	7.4	0.2
6174	3.8	15.9	0.3
7404	6.7	30.5	0.4
8634	9.3	52.6	0.6
9864	13.2	84.5	0.8

Table 4: Wall time for non-iterative procedures of MLHF/aug-cc-pVDZ calculations on the linear amino acid chain, expressed in seconds.

#AOs	$t^{S \text{ Cholesky}}$	$t^{F^{\text{SAD}} \text{ diagonalization}}$	$t^{\text{active MOs}}$
2112	0.3	1.2	0.1
4183	1.4	4.8	0.1
6254	3.3	14.7	0.3
8325	5.8	33.7	0.6
10396	9.2	73.5	0.8
12467	15.3	131.3	1.3
14538	25.6	227.4	1.4
16609	37.9	353.5	2.2

Table 5: Wall time comparison for the MLHF/aug-cc-pVDZ/STO-3G calculations on water clusters of increasing radius, expressed in seconds, with $\boldsymbol{C}$-screening.

	$\boldsymbol{G}(\boldsymbol{D}^{\text{SAD}})$			$\boldsymbol{G}(\boldsymbol{D}^{\text{e}})$			$\boldsymbol{G}(\boldsymbol{D}^{\text{a}})$		
#AOs	t_{pre}	$t_{\mathscr{C}}$	$t_{\mathscr{E}}$	t_{pre}	$t_{\mathscr{C}}$	$t_{\mathscr{E}}$	t_{pre}	$t_{\mathscr{C}}$	$t_{\mathscr{E}}$
958	0.03	4	1	0.03	14	3	0.02	6	2
1889	0.1	21	2	0.1	38	4	0.1	9	3
3751	0.3	88	6	0.2	97	5	0.2	10	3
5606	1	214	10	0.4	145	5	0.3	9	3
7468	1	407	15	1	204	5	1	9	3
9323	2	640	21	1	265	6	1	9	3
11178	3	956	25	1	330	6	1	9	3
13040	4	1313	31	2	392	6	1	9	3
14895	5	1785	37	2	456	6	2	9	3

Table 6: Wall time comparison for the MLHF/aug-cc-pVDZ/cc-pVDZ calculations on water clusters of increasing radius, expressed in seconds, with $\boldsymbol{C}$-screening.

	$\boldsymbol{G}(\boldsymbol{D}^{\text{SAD}})$			$\boldsymbol{G}(\boldsymbol{D}^{\text{e}})$			$\boldsymbol{G}(\boldsymbol{D}^{\text{a}})$		
#AOs	t_{pre}	$t_{\mathscr{C}}$	$t_{\mathscr{E}}$	t_{pre}	$t_{\mathscr{C}}$	$t_{\mathscr{E}}$	t_{pre}	$t_{\mathscr{C}}$	$t_{\mathscr{E}}$
3185	0.2	53	7	0.1	138	24	0.1	11	3
6377	1	255	37	0.5	460	48	0.4	17	4
12761	3	1193	153	2	1360	97	2	25	4
19121	7	3009	318	3	2390	142	3	132	27
25505	13	5664	487	5	3611	189	7	143	28

Table 7: Wall time comparison for the MLHF/aug-cc-pVTZ/cc-pVDZ calculations on water clusters of increasing radius, expressed in seconds, with $\boldsymbol{C}$-screening.

	$\boldsymbol{G}(\boldsymbol{D}^{\mathrm{SAD}})$			$\boldsymbol{G}(\boldsymbol{D}^{\mathrm{e}})$			$\boldsymbol{G}(\boldsymbol{D}^{\mathrm{a}})$		
#AOs	t_{pre}	$t_{\mathscr{C}}$	$t_{\mathscr{E}}$	t_{pre}	$t_{\mathscr{C}}$	$t_{\mathscr{E}}$	t_{pre}	$t_{\mathscr{C}}$	$t_{\mathscr{E}}$
3236	0.2	58	8	0.2	172	40	0.1	83	34
6428	1	265	40	0.5	566	68	0.4	146	46
12812	4	1345	188	2	1584	124	2	234	57
19172	8	3332	390	3	2718	166	3	248	47
25556	15	6548	600	5	4233	228	7	315	57
